# Case Study: Genetic and In Silico Analysis of Familial Pancreatitis

**DOI:** 10.3390/genes16050603

**Published:** 2025-05-20

**Authors:** Yash Sharma, Deborah J. Good

**Affiliations:** 1 Department of Biochemistry, Virginia Tech, Blacksburg, VA 24060, USA; yashsharma@vt.edu; 2 Department of Human Nutrition, Foods, and Exercise, Virginia Tech, Blacksburg, VA 24060, USA

**Keywords:** cystic fibrosis transmembrane receptor gene, chymotrypsin gene, genetic testing, polygenic inheritance, ligand binding domains, HLA genotype

## Abstract

Background/Objectives: Chronic pancreatitis (CP) is a progressive inflammatory condition of the pancreas that leads to irreversible changes in pancreatic structure. The pancreatic α and β cells secrete hormones such as insulin and glucagon into the bloodstream. The pancreatic acinar cells secrete digestive enzymes that break down macromolecules. When these digestive enzymes do not function properly, maldigestion, malabsorption, and malnutrition may result. Presented here is a case study of an individual newly diagnosed with chronic pancreatitis, along with a genetic analysis of his son and an in-silico analysis of two of the variant proteins. Methods: This study was conducted using human subjects, namely, the proband (father) and his son. Medical genetic testing of the proband (father) identified the presence of two variants in the cystic fibrosis transmembrane receptor gene (*CFTR*): variant rs213950, resulting in a single amino acid change (p. Val470Met), and variant rs74767530, a nonsense variant (Arg1162Ter) with known pathogenicity for cystic fibrosis. Medical testing also revealed an additional missense variant, rs515726209 (Ala73Thr), in the *CTRC* gene. Cheek cell DNA was collected from both the proband and his son to determine the inheritance pattern and identify any additional variants. A variant in the human leukocyte antigen (rs7454108), which results in the HLA-DQ8 haplotype, was examined in both the proband and his son due to its known association with autoimmune disease, a condition also linked to chronic pancreatitis. In silico tools were subsequently used to examine the impact of the identified variants on protein function. Results: Heterozygosity for all variants originally identified through medical genetic testing was confirmed in the proband and was absent in the son. Both the proband and his son were found to have the DRB1*0301 (common) haplotype for the HLA locus. However, the proband was also found to carry a linked noncoding variant, rs2647088, which was absent in the son. In silico analysis of variant rs213950 (Val470Met) in CFTR and rs515726209 (Ala73Thr) in CTRC revealed distinct changes in predicted ligand binding for both proteins, which may affect protein function and contribute to the development of CP. Conclusions: This case study of a proband and his son provides additional evidence for a polygenic inheritance pattern in CP. The results also highlight new information on the role of the variants on protein function, suggesting additional testing of ligand binding for these variants should be done to confirm the functional impairments.

## 1. Introduction

Long-term inflammation of the pancreas can cause irreversible damage, leaving the individual with chronic pancreatitis (CP). This condition results in pancreatic fibrosis, leading to both endocrine and exocrine pancreatic insufficiency [1]. Quality of life and life expectancy are both impacted, as the condition can lead to malabsorption, diabetes, and pancreatic cancer. While the development of CP is associated with alcohol use, tobacco use, autoimmune conditions, and certain prescription medications, genetic variants may also predispose individuals to recurrent episodes of pancreatic inflammation, leading to a diagnosis of CP [1]. Recent estimates suggest that up to 73 in 100,000 adults in the U.S. have CP [2].

**Figure 1:**
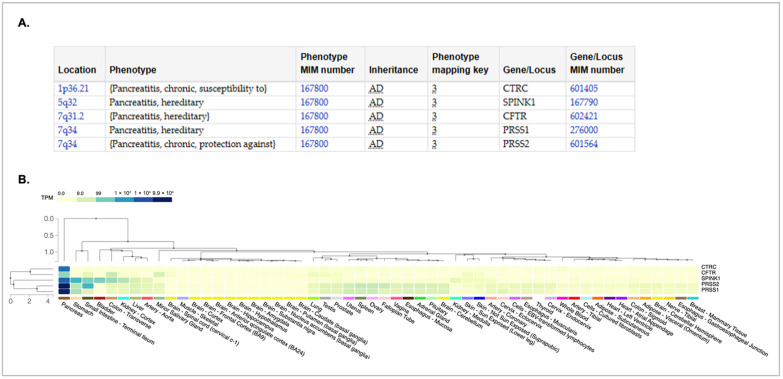
Genes associated with development of hereditary chronic pancreatitis. (**A**) The chart showing the genotype–phenotype relationship was obtained from the Online Mendelian Inheritance in Man (OMIM) database [4,5]. The location of each gene on the chromosome is provided. The inheritance pattern for all are autosomal dominant (AD) The OMIM gene number and phenotype numbers are provided. (**B**) A GTEX multi-gene expression query [6] was used to examine the expression of genes listed in (**A**). The pattern was sorted from the tissue with the highest expression (pancreas) to the lowest (breast/mammary). These data were obtained from the GTEX portal [6] on 4 April 2025.

A recent analysis concluded that routine genetic testing should be conducted for all suspected cases of CP, regardless of age or medical history [3]. According to data from the Online Mendelian Inheritance in Man (OMIM) database [4], variants in five genes have been implicated in the development of chronic pancreatitis (Figure 1A). Each of the known variants is inherited in an autosomal dominant fashion; therefore, heterozygous carriers have an increased predisposition to the disease. Additionally, each of these genes is expressed in the pancreas (Figure 1B). *CTRC* encodes the chymotrypsin C protein, a member of the peptidase S1 protein family that activates and regulates pancreatic enzymes. *SPINK1* encodes the serine peptidase inhibitor Kazal type 1 protein, which is a trypsin inhibitor and is secreted by pancreatic acinar cells. *CFTR* encodes the cystic fibrosis transmembrane regulator and is best known for its association with cystic fibrosis, a condition characterized by impaired water and ion secretion. The CFTR protein is a chloride channel and a member of the ATP-binding cassette transporter superfamily. *PRSS1* and *PRSS2* encode proteins that are members of the trypsin family of proteases, with *PRSS1* encoding trypsinogen and *PRSS2* encoding anionic trypsinogen. Both proteins are highly expressed in the pancreas and are cleaved by chymotrypsin to produce active trypsin—an enzyme essential for protein digestion in the small intestine.

This study sought to characterize the pattern of inheritance in a family with a recent diagnosis of CP. In particular, medical testing was unable to determine if the variants in *CFTR* were in cis or trans. Furthermore, the offspring of this family had not been tested for possible predisposition to CP. Following genetic analysis, in silico tools were employed to further characterize two of the identified missense variants and to better understand their potential role in the etiology of CP.

## 2. Materials and Methods

### 2.1. Human Subjects Determination and Release of Medical Testing Data

The study was approved as expedited by the Virginia Tech Human Research Protection Program. The proband subsequently released their medical testing data to the research team for analysis. The proband and his son provided informed consent for DNA sample isolation and analysis. No other family members were included in this study.

### 2.2. DNA Isolation and Sequencing

The proband and his son provided saliva samples in buffer, which were mixed with a DNA stabilization buffer and stored at 4 °C. DNA was isolated and purified from 2.5 mL samples of saliva donated by each participant using PrepIT-L2P reagent according to the manufacturer’s protocol (DNA Genotek, Ottawa, ON, Canada). The concentration of DNA was evaluated using a NanoDrop spectrophotometer (Thermo-Fisher Scientific, Waltham, MA, USA), and DNA was stored in solution at 4 °C.

Primers were prepared to amplify the surrounding sequence of each variant (Supplemental Table S1) and, following standard PCR amplification, were purified using the GeneJet DNA purification kit (Thermo-Fisher Scientific, Waltham, MA, USA). The PCR fragments were sequenced using both the forward and reverse primers at the Virginia Tech Biocomplexity Institute Genomics Sequencing Center (Blacksburg, VA, USA), and analyzed using the Teal Trace Analysis Viewer (https://www.gear-genomics.com/teal/, accessed on 20 April 2025) [7].

### 2.3. Identification of SNVs and Sequences for Further Study

Each of the variants reported in the patient’s medical report was identified using the search function in the National Center for Biotechnology Information (NCBI) dbSNP database [8], along with the gene name. Sequence data for the genes of interest were obtained from the NCBI protein database [9]. Protein sequences were modified by replacing the reference amino acid with the variant amino acid for further study.

### 2.4. GTEX Gene Expression Analysis

GTEX multiple gene expression portal analysis was used to compare the expression patterns of the proteins [6]. The gene names were entered into the search bar on the GTEX multi-gene query box with all tissue types selected. The data showed the levels of the mRNA from each gene in tissues from the database and were sorted from lowest to highest expression.

### 2.5. Phylogenetic Alignment Analysis

Phylogenetic alignment was conducted using the Clustal Omega Multiple Sequence Alignment program, version 2022 [10] by inputting the normal and variant human sequences, along with the protein sequences from *Macaca mulatta* (Rhesus monkey), *Pan troglodytes* (chimpanzee), *Sus scrofa* (pig), *Bos taurus* (cattle), *Ovis aries* (sheep), *Canus lupis familiaris* (domestic dog), *Felis catus* (domestic cat), *Mus musculus* (mouse), *Rattus norvegicus* (Norway rat), *Gallus gallus* (chicken), *Danio rerio* (zebrafish), and *Xenopus tropicalis* (tropical clawed frog). The output was set to ClustalW with character counts, using default settings for all other parameters. The surrounding sequence of each protein variant was used for comparative analysis.

### 2.6. Tertiary Protein Structure and Ligand Binding Prediction

The IntFOLD7 server [11] was used to generate 3D structural models of the reference and missense variant proteins. Within the IntFOLD7 server interface, the FunFold2 server [12] was used to predict the ligand-binding domains for both the reference and variant proteins [11,13]. PDB files were downloaded from the IntFOLD and FunFold servers and used in the PBD Mol* 3D Viewer [14] to analyze both the structure and ligand binding.

### 2.7. Protein Stability Prediction

I-Mutant 2.0 [15], (https://folding.biofold.org/i-mutant/i-mutant2.0.html, accessed on 20 April 2025) and Mupro1.1 (http://mupro.proteomics.ics.uci.edu/) [15,16] were both used for protein stability prediction. The WT and variant protein sequence information was entered as directed by the two different sites. The scores and instability predictions generated by the sites were reported.

## 3. Results

### 3.1. Medical Genetic Testing Results

Variants identified by the medical genetic testing laboratory in the proband are shown in Table 1. The nonsense variant rs74767530 in CFTR results in a termination of the amino acid chain at position 1162 and is considered pathogenic for cystic fibrosis according the ClinVar classification [17]. No link to pancreatitis has been published. The VarSome composite score [18] also supports a pathogenic/strong likelihood for disease, although it does not specify the type of disease. As our study focused on protein coding regions of the genes, we did not study the rs1042077 variant further.

The proband carried two other CFTR variants: rs1042077, a synonymous variant, and rs213950, a missense variant of Val470Met. The Val470Met variant has previously been associated with acute pancreatitis [19]. One study showed that carriers of the rs1042077 variant who were also homozygous for the more common F508del cystic fibrosis variant exhibited extreme levels of chloride ion in sweat and reduced lung function compared to non-carriers, whereas carriers of rs213950 Val470Met showed no such changes.

Both of the variants identified in PRSS1 in the proband were also synonymous variants (Table 1) and were not studied further. However, a variant in the CTRC gene, rs515726209, results in an Ala73Thr change, and while ClinVar classifies it as “conflicting classifications of pathogenicity” [17], the VarSome composite score and likelihood ratios both classify it as pathogenic [18]. Only one published study has described this variant in patients with idiopathic chronic pancreatitis [20], and no studies of the effects of this variant on protein structure have been published to date.

### 3.2. Pedigree and Analysis of Genomic Locations of Variants

**Figure 2:**
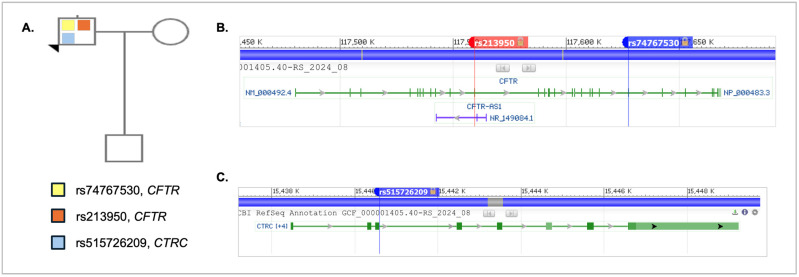
Family pedigree and variant positions in genome. (**A**) The proband is indicated by the arrowhead and is shown as a carrier of the nonsense *CFTR* variant rs747530, the missense *CFTR* variant rs213950, and the missense *CTRC* variant rs515262209. (**B**) The location of the two variants in *CFTR*, rs213950 and rs74767530. (**C**) The location of the rs515726209 in *CTRC*. The data in (**B**,**C**) were obtained using the NCBI SNP database genome variant viewer [17,22].

The proband is a 53-year-old male of South Asian (India) descent who was referred for genetic testing due to a family history and personal diagnosis of pancreatitis. Testing was performed using the Ambry pancreatitis testing panel, which employs sequencing analysis to test for variants in *PRSS1*, *CPA1*, *SPINK1*, *CTRC*, *CASR*, and *CFTR.* Figure 2A shows the pedigree of the proband (indicated by the arrowhead), his wife, and their son. The proband is heterozygous for all variants detected. The pathogenic variant (Arg1162Ter, rs74767530) and missense variant (Val470Met, rs213950) in *CFTR*, and the missense variant in *CTRC* (Ala73Thr, rs515726209) are shown in the pedigree. No additional pathogenic mutations or variants were detected in the other genes on the panel, and the *PRSS1* variants detected are synonymous variants. Figure 2B shows the positions of both variants in *CFTR*. Note that rs74767530 is located in the coding region of exon 22 of the *CFTR* gene and results from a C to T substitution at nucleotide position 3484 in the mRNA (NM_000492.4). Variant rs213950 is located in exon 11 of the coding region for *CFTR*, and results from a G-to-A substitution at nucleotide 1408 in the mRNA (NM_000492.4). Interestingly, this variant is also within the CFTR-AS1 long noncoding RNA (NR_149084.1) called BGas [21]. It is not clear whether the variant would affect the function of BGas in controlling *CFTR* gene expression. Figure 2C shows the position of rs515726209 in exon 3 of the *CTRC* gene (NM_007272.3). This variant results in a G-to-A substitution at nucleotide 217 in the mRNA.

### 3.3. Sequence Analysis of the Proband and Son

**Figure 3:**
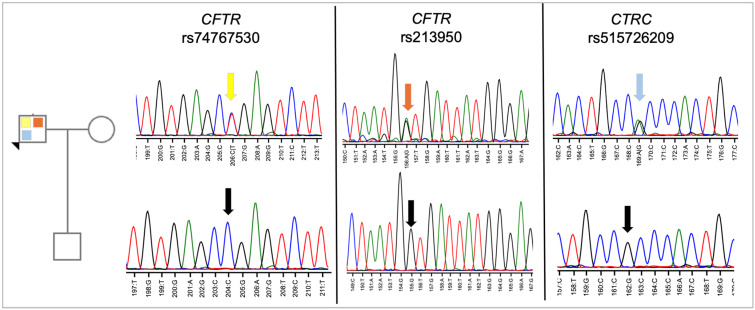
Sequence analysis of proband and son. DNA from the proband and his son was amplified by PCR using flanking primers and then sequenced using the forward primer. As shown, the proband is heterozygous for each variant, while his son carries only the reference sequence. The chromatogram is colored by nucleotide, with T (thymidine) = red, G (guanine) = black, A (adenine) = green, and C (cytosine) = blue. The colored arrows on the sequence from each gene points to the heterozygous nucleotide at that position. The color code directly relates to the variant color shown in the pedigree. The black arrows in the sequence indicate the reference sequence.

Human subject approval was obtained from the Virginia Tech Institutional Review Board. The proband and his son provided informed consent and agreed to undergo genetic testing in our research laboratory. Both provided saliva samples for DNA isolation. The mother/wife was not asked to provide a DNA sample nor was consent necessary to obtain. DNA from each region of the indicated genes was amplified using primers designed by the laboratory (Supplemental Table S1, Supplemental Figure S1), and DNA was then purified for sequence analysis. As shown in Figure 3, the proband was confirmed to be heterozygous for all three variants, while the son was found to carry none of the variants. The absence of both CFTR variants in the proband’s son suggests that the two CFTR variants are in cis in the proband, on the same chromosome, as the two variants are only 68,0058 nucleotides apart, and crossover events would be less likely. However, our results do not rule out the possibility of a crossover event during gametogenesis in the proband. Given the lack of variants identified in the son, his heritability of CP is low.

**Figure 4:**
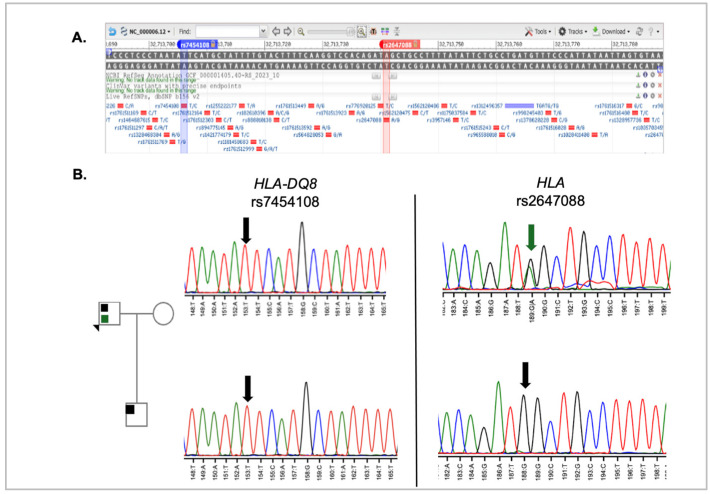
HLA locus analysis in proband and son. (**A**) SNP position in HLA locus. Data was captured from NCBI SNP database genome variant viewer for rs7454108 and rs2647088. [17,22]. The blue bar indicates the position of rs7454108 in the HLA gene, while the red bar indicates the position of rs2647088 in the HLA sequence (**B**) Sequence analysis results for HLA-DQ8 rs7454108 and HLA rs2647088. The green colored arrow on the HLA sequence points to the heterozygous nucleotide at that position. The color code direct relates to the variant color shown in the pedigree. The black arrows in the sequence indicate the reference sequence. The nucleotides in the chromatogram are colored as described in Figure 3.

Autoimmune pancreatitis accounts for up to 11% of chronic pancreatitis cases [23]. Studies have associated celiac disease, which often presents with the HLA-DQ8 genotype, with nearly a threefold increase in the incidence of autoimmune pancreatitis [24]. In a mouse model carrying the human HLA-DQ8 locus, pancreatitis developed following development of celiac disease with gliadin sensitization. For this reason, both the proband and his son were tested for the HLA-DQ8 genotype. As shown in Figure 4, both the proband and son were homozygous for the T allele, which is the reference genotype (i.e., not HLA-DQ8). However, the father is heterozygous for rs2647088, which is located 36 base pairs downstream. As there are no publications referencing this variant, the implication of this finding remains unknown.

### 3.4. Allele Frequencies for Tested Variants

The NCBI SNP database population frequencies were used to produce Table 2*,* which shows the global allele frequency and Asian allele frequency for all variants assessed in this study. As shown, the proband was heterozygous for each of the minor alleles tested in the *CFTR* and *CRTC* genes. For *CFTR* rs74767530, the allele frequency for the T allele in Asians is 0, compared to the global allele frequence of 0.00086, indicating that this allele and genotype is quite rare. For the *CFTR* rs213950 variant, the allele frequencies of the reference and variant alleles are closer in both global and Asian populations. The *CTRC* rs51526209 has two variant alleles in addition to the reference allele. Our proband carries the A allele in combination with the reference G allele. The A allele is rare in both the global and Asian populations. In testing for the *HLA-DQ8* genotype, both the proband and his son were found to carry the reference allele. However, a variant was found in the proband just downstream of the variant tested for *HLA-DQ8.* This G allele is relatively common both globally and within Asian populations, being found in 27–37% of individuals.

### 3.5. Phylogenetic Analysis of Missense Variants

**Figure 5:**
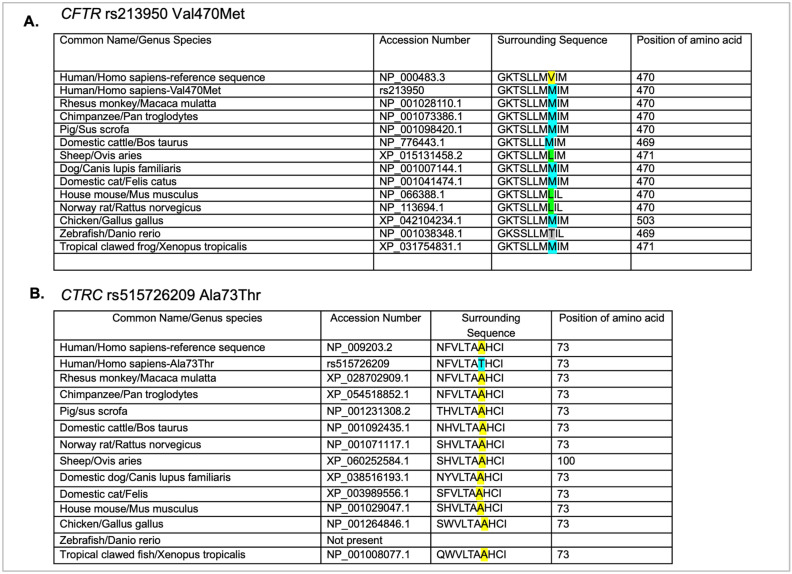
Phylogenetic analysis of the missense variants in CFTR (**A**) and CTRC (**B**). Common name, genus species name, accession number, sequence, and position of amino acid in the ortholog from each species are shown. Yellow highlighted amino acids indicate the amino acid in the reference sequence for that position. The blue highlight indicates the variant amino acid. The green amino acids in panel A indicate a different variant amino acid at that position.

Phylogenetic analysis of protein sequences can provide insight into amino acid positions within proteins through evolution. We examined the protein sequences for the two missense variants found in the proband’s DNA (Figure 5). The CFTR variant rs213950 changes a valine to a methionine. Surprisingly, methionine residues are more common among the vertebrates examined, while none of the vertebrates examined, including other primates, had a valine residue at that relative position (Figure 5A). Leucine amino acid was present in sheep, mouse, and rat, while threonine was present in the zebrafish CFTR protein sequence. Interestingly, there was little variability in the surrounding amino acids of zebrafish and xenopus, with 100% of the preceding seven amino acids conserved. These findings suggest that methionine may be an ancestral allele, and that homo sapiens evolved a valine at that location for an unknown advantage. To support this hypothesis, Neanderthal DNA was examined using the UCSC genome browsers. As shown in Supplemental Figure S2, Neanderthal CFTR also contains the ATGGTG sequence, which would code for an MV amino acid dyad at that position. Conversely, Denisovan contains the ATGATG sequence, which, like the other primates listed, would code for an MM amino acid dyad at that position.

In examining the amino acid sequence for CTRC rs51526209 (A73T), the reference alanine amino acid was conserved through all vertebrates examined, with the exception of zebrafish, which currently do not have a known chymotrypsin gene (Figure 5B). However, in the case of CTRC, there was more variability in the preceding six amino acids, and the preceding three amino acids examined were 100% conserved. These findings suggest that the substitution of threonine for alanine has the potential to change the function of the protein.

### 3.6. Tertiary Structure of Proteins with Ligand Binding Analysis

In silico tools were used to examine predicted stability changes, tertiary structure changes, and predicted changes in ligand binding. For protein stability analysis, the amino acid sequences of CTRC and CFTR were input into two computational tools: I-Mutant 2.0 [15] and Mupro1.1 [15,16]. As shown in Supplemental Table S2, both programs predicted decreased overall stability for the missense variants in CTRC and CFTR, based on a reduction in the delta delta G free energy within the variant sequence.

#### 3.6.1. CFTR Reference Protein and rs213950 Val470Met Variant

**Figure 6:**
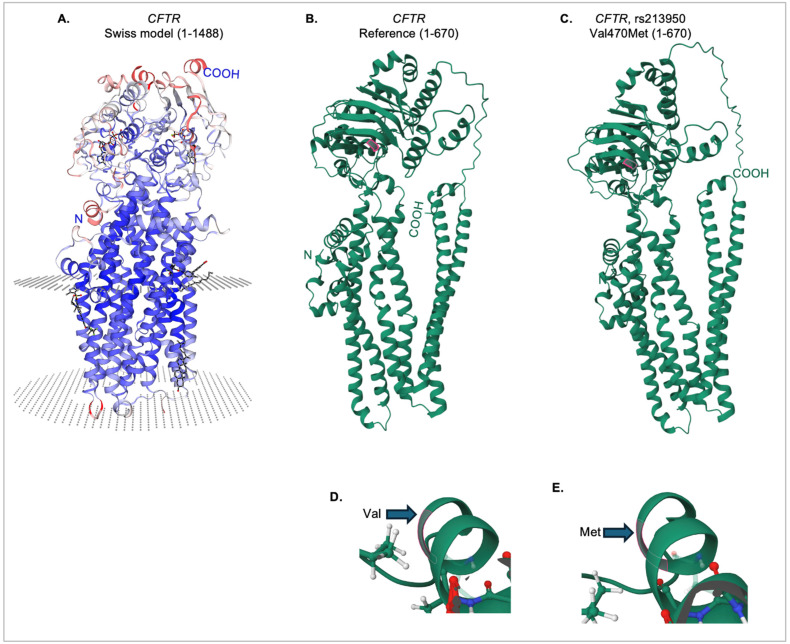
Tertiary structure of the reference and Val470Met CFTR proteins. (**A**) Swiss-Model [25] CFTR protein model, showing the predicted membrane-embedded regions (circular dotted areas) for the receptor (amino acids 1-1488). (**B**) IntFOLD7 predicted tertiary structure for amino acids 1-670 of the reference CFTR protein. The valine at position 470 is highlighted in pink. (**C**) IntFOLD7 predicted tertiary structure for amino acids 1-670 of the rs213950 variant protein. The methionine at position 470 is highlighted in pink. Close-up view of the tertiary structure near the valine 470 in the reference protein (**D**), and the methionine 470 in the variant protein (**E**).

As shown in Figure 6A, Swiss-Model [25] was used to obtain a predicted model of a nearly complete CFTR sequence (amino acids 1-1488), with prediction of the membrane interaction domains. The amino (N) and carboxy (COOH) ends of the protein chain are indicated. The IntFOLD7 server was then used to generate predicted models for shorter chains of both the reference and variant protein sequences, as there are limitations on the size of input for this server. However, as shown in Figure 6B, the reference protein model was consistent in overall structure, missing only an extracellular domain from the C-terminus. The valine at position 470 is outlined (Figure 6D). Likewise, the same sequence with the variant methionine residue (outlined, Figure 6E) was input into the IntFOLD7 server, and the resultant 3D structure was consistent with the CFTR protein (Figure 6C).

**Figure 7:**
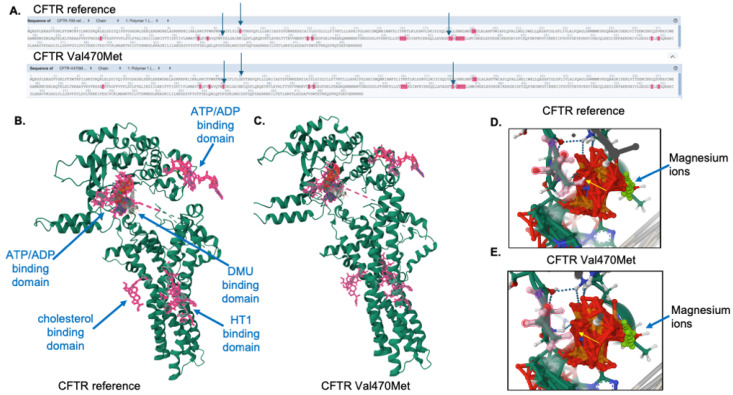
Ligand binding analysis for the reference and variant CFTR proteins. (**A**) Predicted ligand-binding amino acid interactions are shown in pink highlights. Arrows indicate ligand binding differences between the reference and variant proteins. This figure was generated and edited from the FunFold software. (**B**) The CFTR reference protein with ligands are indicated by pink residues and labeled. (**C**) The Val470Met CFTR protein with ligands are indicated by pink residues. The threonine 460 amino acid residue is highlighted in pink for the reference (**D**) and variant (**E**) CFTR proteins. The arrow indicates the predicted loss of interaction with the ATP/ADP binding domain.

FunFold analysis, which is part of the IntFOLD7 server, was used to predict ligand binding domains for the reference and variant proteins. As shown in Figure 7A, there is a loss of two ligand-binding interactions in the variant protein at positions 92 (E, glutamine) and 460 (T), as indicated by the absence of pink highlights from the sequence, as well as the arrows. In addition, there is a gain of ligand-binding interaction in the variant protein in position 362 (Y, tyrosine). The full structures of both the reference (Figure 7B) and the Val470Met variant proteins (Figure 7C) are shown, with all ligands indicated by pink structures and labels. The variant protein is predicted to interact with each of the ligands that are predicted for the reference protein. The E92 position interacts with the HT1 ligand, which is predicted to be a Hoechst 33342 dye binding site, though the significance of that interaction is unknown. The Y362 position, which shows a gain of ligand binding for the variant protein, is within the interaction site for cholesterol. Cholesterol interaction with the membrane-embedded portion of the CFTR protein [26,27] and alterations in cholesterol metabolism have been previously shown for variants causing cystic fibrosis [28]. Figure 7D,E illustrate a loss of interaction with ATP/ADP at position 460 (threonine) in the Val470Met variant protein, compared to the reference CFTR protein. This position lies just 10 amino acids away from the variant methionine residue and falls within the ATP/ADP binding site. Although the predicted magnesium ion binding site, as well as the ATP/ADP interaction, are still preserved, the effect of this predicted loss at T460 remains unknown.

#### 3.6.2. CTRC Reference and rs213950 Variant

**Figure 8:**
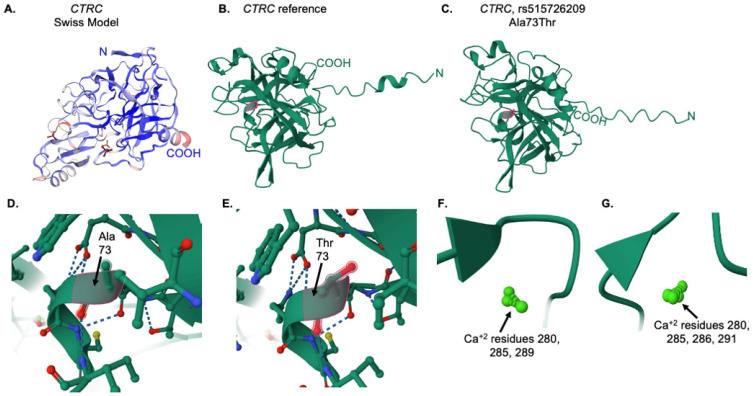
Tertiary structure and ligand analysis for the CTRC reference and variant proteins. (**A**) Swiss-Model [25] predicted structure for the reference CTRC protein. (**B**) IntFOLD7 predicted structure for the reference CTRC protein. The alanine residue at position 73 is highlighted in pink. (**C**) IntFOLD7 predicted structure for the A73T variant CTRC protein. The threonine residue at position 73 is highlighted in pink. (**D**) Close-up view of the alanine at position 73 in the reference protein, as indicated by the pink outline and arrow. (**E**) Close-up view of the threonine at position 73 in the variant protein, as indicated by the pink outline and arrow. In (**D**,**E**), the dotted lines represent ionic interactions between amino acids (**F**) Calcium ions in the reference CTRC protein. (**G**) Calcium ions in the variant protein.

Swiss-Model [25] was used to obtain a predicted model of the complete CTRC sequence (Figure 8A). The amino (N) and carboxy (COOH) ends of the protein chain are indicated. The IntFOLD7 server was then used to generate predicted models for both the reference and variant protein sequences. As shown in Figure 8B, the reference protein model predicted by IntFOLD7 was consistent in overall globular structure, with slight differences in the N- and C-terminal regions. The alanine at position 73 in the reference protein is highlighted in pink (Figure 8B). Likewise, the same sequence with the variant threonine residue at position 73 was input into the IntFOLD7 server, and the resultant 3D structure is consistent with the CTRC protein (Figure 8C), with some notable differences in the loss of an α-helical structure at the N-terminus. A closer view of the local region for alanine 73 (Figure 8D) and threonine 73 (Figure 8E) are shown. Using the FunFold program as part of the IntFOLD7 server, predicted ligands were analyzed. While there were no differences in amino acid interaction with all predicted CTRC ligands, which included PBZ (p-amino benzamidine), FUL (β-L-fucopyranose), and TFI (an aniline drug compound; a difference was noted in the number of bound calcium molecules between the reference and variant CTRC proteins. As shown in Figure 8F,G there are three calcium molecules (numbered 280, 285, and 289 by the prediction software) in the reference CTRC protein, but four calcium molecules (numbered 280, 285, 286, and 291) in the Ala73Thr variant protein. Calcium binding is essential for CTRC protein interaction [29]. The effect of this change in calcium ion number within the variant protein remains unknown.

## 4. Discussion

In this case study of an individual with chronic pancreatitis, analysis of DNA from the son provided additional genetic insight that not only alleviated concerns surrounding inheritance of the deleterious variants but also provided some information about which alleles of *CFTR* in the proband carried the variants. The fact that the son has none of the tested variants for *CFTR* suggests that both are carried on a single allele, in cis, rather than one of each allele in trans. The nonsense variant rs74767530 lies downstream of the missense variant rs213950, likely creating a completely non-functional *CFTR* allele that carries both variants. These data are consistent with a study of 134 patients, which reported that all CP patients were heterozygous for *CFTR* variants [30]. Interestingly, rs213950 is located within an antisense noncoding RNA locus contained within the *CFTR* gene, known as *CFTR-AS1*, also referred to as BGas [21]. This noncoding RNA appears to modulate and reduce expression of CFTR mRNA [21]. The transcript appears to contain two exons which, when spliced, produce a 240-nucleotide long noncoding RNA, with rs213950 lying within the second intron. However, if the rs213950 variant functionally inactivates BGas/*CFTR-AS1*, then we might expect upregulation of the normal allele of the *CFTR* gene in the proband; however, additional testing is required to confirm this hypothesis.

HLA genotyping of the proband and his son revealed that neither carried the DQ8 genotype, which has been linked to autoimmune pancreatitis [23,31]. However, a rare variant, rs2647088, was detected in the proband but not in his son. As there are no known disease associations for this variant, no conclusions can be drawn regarding its potential role in carriers.

Individuals who are homozygous for the nonsense variant rs74767530 are typically monitored for symptoms of cystic fibrosis (CF), as this variant is a known single genetic cause of CF in multiple patients (e.g., [32,33]). In one study, all nine patients who were homozygous for rs74767530 exhibited mild to moderate CF along with pancreatic insufficiency. Although pancreatic insufficiency can occur alongside a diagnosis of pancreatitis, it can also arise from entirely different etiologies. Our proband was heterozygous for rs74767530, a status consistent with individuals who present with CP but not CF [30].

We show that the proband carries missense variants in two different genes implicated in the etiology of CP—*CFTR* and *CTRC*—as well as a nonsense variant in *CFTR*, which may affect the same allele as the missense variant in that gene. With the availability of CP panels from commercial laboratories, a recent study examined monogenic and polygenic CP diagnoses. Among the 631 cases, only 8.6% had a clinically significant panel result, the majority of which involved monogenic *CFTR* variants, followed by variants in *SPINK1* and *PRSS1* [3]. Monogenic variants in *CTRC* accounted for only 1.6% of cases with a positive genetic outcome, while polygenic cases constituted 1.7% of all identified cases, including those having variants in both *CTRC* and *CFTR* [3]. These findings suggest that there is a possible additive effect from the inheritance of two variants; however, further research is needed to confirm this hypothesis.

While the nonsense variant in *CFTR* is classified as pathogenic for cystic fibrosis in ClinVar [17], the other *CFTR* missense variant in the proband has a classification of benign/likely benign. However, the VarSome score [18] provides support for pathogenicity, and our in silico findings may have identified the role of rs213950 in pathogenicity. Although the structural change resulting from the Val470Met substitution appears visually minimal, the change occurs near several ligand-binding regions of CFTR, including one of its ATP/ADP binding domains. To date, no further analysis has been conducted on the effect of rs213950 on ATP binding; however, this would be a logical next step in understanding the impact of the variant. In our study, we also identified two additional predicted changes in ligand-binding interactions involving the HT1 ligand, which is predicted to be a Hoechst 33342 dye interaction site at position E92. Although no published studies have examined interactions between CFTR Val470Met and Hoechst dyes, research on a related protein, P-glycoprotein, has shown that Hoechst 33342 can promote the maturation and folding of variant proteins [34,35]. Folding of several CFTR missense variants can be improved using ligands such as VRT-325, a small-molecule ligand [35]. However, to our knowledge, Hoechst 33342 dye has not been tested with Val470Met or other CFTR variants. The ligand-binding analysis also identified a gain of ligand binding for cholesterol at the Y362 position in the Val470Met variant. While increased cholesterol accumulation has been observed in CFTR knockout mice [28], the Val470Met variant—with its potential gain in cholesterol binding—has not yet been tested.

The missense variant in *CTRC*, rs515726209 (Ala73Thr), identified in the proband through both clinical testing and our study, has been given conflicting classifications of pathogenicity in ClinVar [17], and is rated as pathogenic/strong by the VarSome composite score [18]. There is only one citation for this rare variant in ClinVar, which only describes its prevalence in patients with pancreatitis [20]. However, another study demonstrated that the Ala73Thr variant prevents secretion of the CTRC protein in both HEK293 cells and a rat pancreatic acinar cell line [36]. In our study, the visible predicted 3D structure of the protein was maintained in the Ala73Thr variant, but ligand binding analysis demonstrated a gain in calcium ion binding from three ions in the WT protein to four ions in the variant protein. Recent studies show that chymotrypsin in colonic epithelial cells is activated by calcium signaling pathways [37]. It is not yet clear how increased calcium interaction might affect the activity of the CTRC protein, but the two mutant stability prediction programs we employed, together with the secretion data from Rosendahl and colleagues [36], suggest that the stability of the variant protein may be compromised. A follow-up study from the same laboratory further demonstrated that the Ala73Thr variant exhibited one of the lowest catalytic activities among all tested variants in their analysis [38].

## 5. Conclusions

This case study of a proband and his son provides additional evidence for a polygenic inheritance pattern in CP, as well as insight into the impact of each identified variant on protein function. The study raises further questions that will require wet laboratory testing, particularly in the area of ligand binding for the variant CFTR and CTRC proteins. New findings on the potential effect of ATP binding in CFTR suggest that existing CFTR drugs may help restore ATP interaction function in carriers of rs213950, as has been demonstrated in other variants for the small molecule CP-628006 [39] and the more common drug Ivacaftor, which stabilizes that domain [40]. Drugs that modulate CTRC activity do not appear to be available for human use in the United States. Oral chymotrypsin is used in burn therapy, and a veterinary formulation of chymotrypsin is available as Kymar Ointment, which also contains hydrocortisone, neomycin, and trypsin for topical usage. It is possible that oral chymotrypsin could be used for carriers of the rs515726209 variant; however, basic research followed by clinical trials would be necessary to evaluate this off-label use.

Overall, the findings of this study open new areas for investigating variants associated with CP, particularly in the analysis of ligand-binding interactions and potential drug treatment strategies. Further studies involving patients with a possible polygenic etiology may also help to elucidate the additive effects of variants on the severity of the condition.

## Figures and Tables

**Table 1:** Analysis of variants detected in proband’s DNA.

Gene	DNA Variation	Amino Acid	Variant Type and SNP Number	ClinVar Classification [17]	VarSome Composite Score [18]	Likelihood Ratio Test Algorithm (VarSome) [18]
*CFTR*	*NM_000492.4:c.3484C>T*	R [CGA] > * [TGA] *Arg1162Ter*	Nonsense Variant *rs74767530* https://www.ncbi.nlm.nih.gov/snp/rs74767530 (accessed on 20 April 2025)	Pathogenic for cystic fibrosis	Pathogenic/strong	Uncertain
*CFTR*	*NM_000492.4:c.2562T>G*	T [ACT] > T [ACG] *Thr854=*	Synonymous Variant *rs1042077* https://www.ncbi.nlm.nih.gov/snp/rs1042077 (accessed on 20 April 2025)	Benign/Likely Benign	Benign/strong	N/A
*CFTR*	*NM_000492.4:c.1408G>A*	V [GTG] > M [ATG] *Val470Met*	Missense Variant *rs213950* https://www.ncbi.nlm.nih.gov/snp/rs213950 (accessed on 20 April 2025)	Benign/Likely Benign	Benign/strong	Pathogenic Supporting
*PRSS1*	*NM_002769.5:c.486T>C*	D [GAT] > D [GAC] *Asp162=*	Synonymous Variant *rs6666* https://www.ncbi.nlm.nih.gov/snp/rs6666 (accessed on 20 April 2025)	Benign/Likely Benign	Benign/moderate	N/A
*PRSS1*	*NM_002769.5:c.738T>C*	N [AAT] > N [AAC] *Asn246=*	Synonymous Variant *rs6667* https://www.ncbi.nlm.nih.gov/snp/rs6666 (accessed on 20 April 2025)	Benign/Likely Benign	Benign/strong	N/A
*CTRC*	*NM_007272.3:c.217G>A*	A [GCC] > T [ACC] *Ala73Thr*	Missense Variant *rs515726209* https://www.ncbi.nlm.nih.gov/snp/rs515726209 (accessed on 20 April 2025)	Conflicting classifications of pathogenicityPathogenic (2); Likely pathogenic (1); Uncertain significance (1) for hereditary pancreatitis	Pathogenic/strong	Pathogenic Supporting

Red-nucleotides that are different between WT and variant. Gray-invariant nucleotides. “*”-indicates stop codon gained. N/A—not available. There were none given by the program. “=“—synonymous variant, no amino acid change.

**Table 2:** Allele frequencies for tested variants.

Gene/Variant	Global Allele Frequency	Asian Allele Frequency
*CFTR* *rs74767530*	Sample size: 210,486 C = 0.999914 T = 0.000086	Sample size: 6592 C = 1.0000 T = 0.0000
*CFTR* *rs213950*	Sample size: 361,510 G = 0.577063 A = 0.422937	Sample size: 6928 G = 0.5592 A = 0.4408
*CTRC* *rs515726209*	Sample size: 30,086 G = 0.99997 T = 0.00000 A = 0.00003	Sample size: 202 G = 1.000 T = 0.000 A = 0.000
*HLA-DQ8* *rs7454108*	Sample size: 223,290 T = 0.898303 C = 0.101697	Sample size: 6598 T = 0.9230 C = 0.0770
*HLA* *rs2647088*	Sample size: 30,272 A = 0.72612 G = 0.27388	Sample size: 436 A = 0.631 G = 0.369

## Data Availability

The raw data supporting the conclusions of this article will be made available by the authors on request.

## Supplementary Materials

The following supporting information can be downloaded at: https://www.mdpi.com/article/10.3390/genes16050603/s1, Figure S1: PCR amplification of genomic DNA from sample 100 and 101; Figure S2: Phylogenetic comparison of the CTRC protein coding sequence from humans, Neanderthals, Denisovans, and other primates. Table S1: Primers used for amplification of regions of interest; Table S2. Effect of variants on protein stability according to I-Mutant 2.0. https://folding.biofold.org/i-mutant/i-mutant2.0.html (accessed on 4 April 2025), and MutPro 1.1 https://mupro.proteomics.ics.uci.edu/ (accessed on 4 April 2025).
