# Supplementary material for: Case Study: Genetic and In Silico Analysis of Familial Pancreatitis
Source: Genes (Basel). 2025 May 20;16(5):603. doi: 10.3390/genes16050603 (PMC12110861; doi:10.3390/genes16050603)
Supplement: Supplementary file 1 [file genes-16-00603-s001.zip › genes-3642607-supplementary.pdf]

Supplemental Figure S1. PCR amplification of genomic DNA from sample 100 (proband) and 101 (son). **A.** *HLA-DQ8* and *CFTR* rs74767530 amplification. **B.** *CFTR,* rs213950 amplification **C.** *CTRC,* rs515726209 amplification.

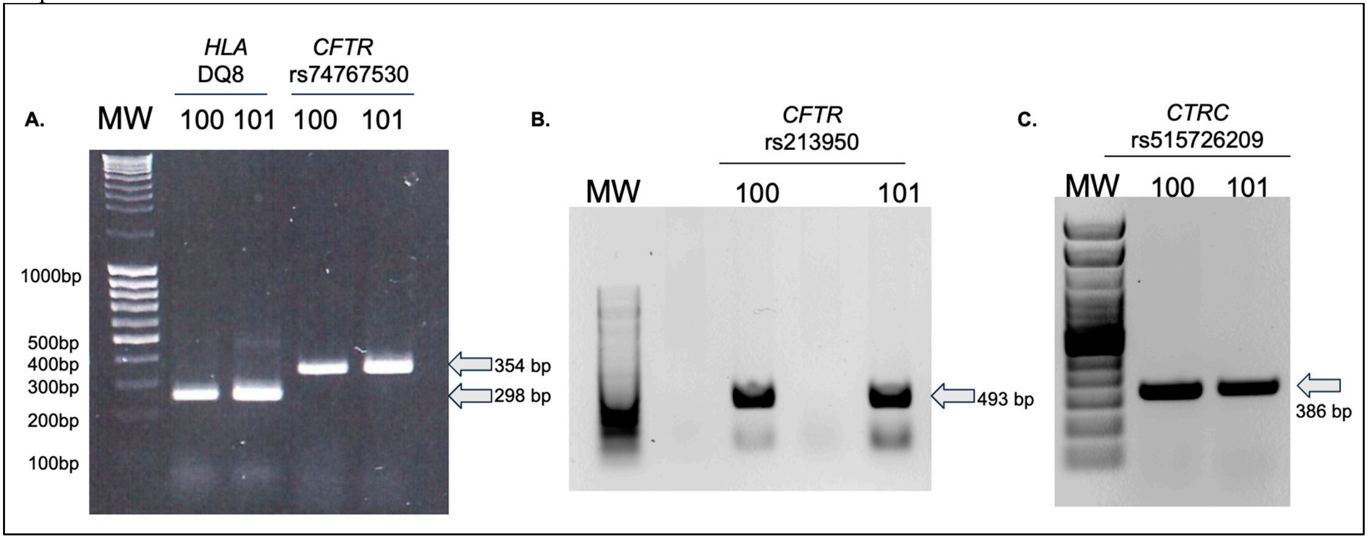

Supplemental Figure S2. Phylogenetic comparison of the CTRC protein coding sequence from Humans, Neanderthal and Denisova and other primates.

```
Human         ttccagacttcacttctaatggtgattatgggagaactggagccttcagagggta
Marmoset      ttccagacttcacttctaatgatgattatgggagaactggagccttcagagggta
Rhesus        ttccagacttcacttctaatgatgattatgggagaactggagccttcagagggta
Orangutan     ttccagacttcacttctaatgatgattatgggagaactggagccttcagagggta
Chimp         ttccagacttcacttctaatgatgattatgggagaactggagccttcagagggta
Mouse lemur   ttccagacttcacttctaatgatgattatgggagaactagagccttcagagggca
Bushbaby      ttccagacttcacttctaatgatgattatgggagaactggagccttcagagggta
Tarsier       tttcagacttcacttctaatgatgattatgggagaactggaacctttagagggta
Neanderthal   ttccagacttcacttctaatggtgattatgggagaactggagccttcagagggta
Denisova      tttcagacttcacttctaatgatgattatgggagaactggaacctttagagggta
```

Supplemental Table S1. Primers used for PCR amplification and sequencing of regions of interest.

**Gene/variant**	**Forward Primer**	**Reverse Primer**	**Amplicon Size**
CFTR/ rs74767530	5′-ACAGGCCTATACAGAGCCCA-3′	5′-AGCAGTGTTCAAATCTCACCCT-3′	493 bp
CFTR/ rs213950	5′-TGTGCATAGCAGAGTACCTGAA-3′	5′-TTGGCATGCTTTGATGACGC-3′	354 bp
CTRC/ rs515726209	5′-AGGTAAGCCTGTGTAGGGCT-3′	5′-AACTGAGTTACTGGGTGTGAGT-3′	333 bp
HLA-DQ8/ rs7454108	5′-GAGACCCCTTTGCAATCT-3′	5′-ATAATGGGAAACATCAGGCAGA-3′	495 bp

Supplemental Table S2. Effect of variants on protein stability according to I-Mutant 2.0. https://folding.biofold.org/cgi-bin/i-mutant2.0.cgi and MutPro 1.1 https://mupro.proteomics.ics.uci.edu/cgi-bin/predict.pl

**I-Mutant 2.0 Stability Prediction**			
**Variant**	**Delta delta G-Free Energy Change Value (kcal/mol)**	**Reliability Index (1-10 with 10 most reliable)**	**Stability**
CFTR rs213950 Val470Met	-1.53	7	Decreased
CTRC rs515726209 Ala73Thr	-1.00	7	Decreased
**MuPro 1.0 Stability Prediction**			
**Variant**	**Delta delta G-Free Energy Change Value (kcal/mol)**	**Confidence Score Smaller = more confident prediction**	**Stability**
CFTR rs213950 Val470Met	-1.07	-0.731	Decreased
CTRC rs515726209 Ala73Thr	-1.64	-0.998	Decreased
